# Noninvasive cardiac-specific biomarkers for the diagnosis and prevention of vascular stenosis in cardiovascular disorder

**DOI:** 10.3389/fphar.2024.1376226

**Published:** 2024-04-25

**Authors:** Sujith Kumar Pulukool, Sai Krishna Srimadh Bhagavatham, Sudarshan K. Vijay, Abdulrahman I. Almansour, Sandeep Chaudhary, Farah Abuyousef, Na’il Saleh, Pratima Tripathi

**Affiliations:** ^1^ Department of Biosciences, Sri Sathya Sai Institute of Higher Learning, Puttaparthi, Andhra Pradesh, India; ^2^ Department of Cardiology, Dr. Ram Manohar Lohia Institute of Medical Sciences, Lucknow, Uttar Pradesh, India; ^3^ Department of Chemistry, College of Science, King Saud University, Riyadh, Saudi Arabia; ^4^ Department of Medicinal Chemistry, National Institute of Pharmaceutical Education and Research (NIPER-R), Lucknow, Uttar Pradesh, India; ^5^ Department of Chemistry, College of Science, United Arab Emirates (UAE) University, Al Ain, United Arab Emirates; ^6^ Department of Biochemistry, Dr. Ram Manohar Lohia Institute of Medical Sciences, Lucknow, Uttar Pradesh, India

**Keywords:** vascular stenosis, coronary artery disease (CAD), high sensitivity troponin I (hs-TnI), cardiac troponin I (c-TnI), high sensitivity C-reactive protein (hs-CRP), single vessel disease (SVD), multiple vessel disease (MVD)

## Abstract

**Background:**

The most frequent lesion in the blood vessels feeding the myocardium is vascular stenosis, a condition that develops slowly but can prove to be deadly in a long run. Non-invasive biomarkers could play a significant role in timely diagnosis, detection and management for vascular stenosis events associated with cardiovascular disorders.

**Aims:**

The study aimed to investigate high sensitivity troponin I (hs-TnI), cardiac troponin I (c-TnI) and high sensitivity C-reactive protein (hs-CRP) that may be used solely or in combination in detecting the extent of vascular stenosis in CVD patients.

**Methodology:**

274 patients with dyspnea/orthopnea complaints visiting the cardiologists were enrolled in this study. Angiographic study was conducted on the enrolled patients to examine the extent of stenosis in the five prominent vessels (LDA, LCX, PDA/PLV, RCA, and OM) connected to the myocardium. Samples from all the cases suspected to be having coronary artery stenosis were collected, and subjected to biochemical evaluation of certain cardiac inflammatory biomarkers (c-TnI, hsTn-I and hs-CRP) to check their sensitivity with the level of vascular stenosis. The extent of mild and culprit stenosis was detected during angiographic examination and the same was reported in the form significant (≥50% stenosis in the vessels) and non-significant (<50% stenosis in the vessels) Carotid Stenosis. Ethical Clearance for the study was provided by Dr. Ram Manohar Lohia Institute of Medical Sciences Institutional Ethical Committee. Informed consent was obtained from all the participants enrolled in the study.

**Results:**

We observed that 85% of the total population enrolled in this study was suffering from hypertension followed by 62.40% detected with sporadic episodes of chest pain. Most of the subjects (42% of the total population) had stenosis in their LAD followed by 38% who had stenosis in their RCA. Almost 23% patients were reported to have stenosis in their LCX followed by OM (18% patients), PDA/PLV (13%) and only 10% patients had blockage problem in their diagonal. 24% of the subjects were found to have stenosis in a single vessel and hence were categorized in the Single Vessel Disease (SVD) group while 76% were having stenosis in two or more than two arteries (Multiple Vessel Disease). hs-TnI level was found to be correlated with the levels of stenosis and was higher in the MVD group as compared to the SVD group.

**Conclusion:**

hs-TnI could be used as a novel marker as it shows prominence in detecting the level of stenosis quite earlier as compared to c-TnI which gets detected only after a long duration in the CVD patients admitted for angiography. hs- CRP gets readily detected as inflammation marker in these patients and hence could be used in combination with hs-TnI to detect the risk of developing coronary artery disease.

## Introduction

The most frequent lesion in the blood vessels feeding the myocardium is vascular stenosis, which has both medicinal and surgical consequences associated with it. Aortic valve sclerosis, or thickening and calcification of the aortic valve without a pressure gradient, appears to affect roughly one-fourth of the elderly and people with certain underlying metabolic conditions linked to hypercholesterolemia. One of the major leading causes of cardiovascular disease (CVD), which is the current leading cause of death and disability worldwide predicted to overtake all other causes of medical mortality by 2030, is vascular stenosis (Yan et al., 2017). The capacity to recognize high-risk people before overt events emerge as an essential criteria for the primary prevention of CVD. This emphasizes the importance of precise risk classification in the patients for a better diagnosis and treatment outcomes. There are several new biomarkers being discovered to predict stenosis and related cardiovascular events. When defining, predicting, and making management decisions for cardiovascular events, biomarkers are essential (Paradis et al., 2014). These describe a broad class of quantitative and repeatable biological sign properties. Multiple artery beds are affected by the systemic inflammatory vascular condition known as stenosis (Ärnlöv et al., 2010). Due to the existence of carotid artery disease in addition to stroke, these individuals are at exceptionally high risk for a variety of atherosclerotic cardiovascular (CV) events, notably coronary events. Patients who have both coronary and peripheral artery disease, which is linked to the occurrence of atherosclerotic disease in various arterial systems, are twice as likely to experience repeated symptoms and consequences as patients who just have coronary artery disease (CAD) (Lind et al., 2022). Even though the prognosis of patients with atherosclerotic vascular disease has significantly improved, the contemporary medication and revascularization procedures are given due credit for treatment of atherosclerosis-related CVD events that continue to account for over 46% of all occurrences of fatalities in affluent nations. Preclinical CAD in patients with substantial carotid artery stenosis may be detected and treated to enhance long-term outcomes and survival (Karelis et al., 2004). Cardiac biomarkers are bio-molecular substances that are extracted through venal punctures and discharged into the blood when the heart is wounded or under stress (Thupakula et al., 2022). Testing for heart biomarkers can help determine a person’s risk of developing cardiac conditions, as well as monitor and treat someone who may be experiencing myocardial ischemia and the acute coronary syndrome (ACS). The identification of high-risk individuals, the rapid and accurate diagnosis of disease states, and the efficient diagnosis and treatment of patients are all made possible by biomarkers (Ahmad et al., 2023). The promptness and sensitivity of hs-TnI and hs-CRP as compared to c-TnI (that had been utilized as a universal marker till date) poses the need of extensive study of these biochemicals to declare them as cardiac biomarkers and to be examined during cardiac inflammations. Imaging techniques such as CT angiography (CTA) is also available as a minimal invasive procedure to detect stenosis in the cardiac vessels. While the biomarkers can be detected by just withdrawing a few milliliters of blood sample from the patient and may be a means to diagnose the level of stenosis even at a very earlier stage, the disadvantages associated with imaging procedures are that they are too expensive and may be associated with side effects like contrast induced nephropathy that could also be life threatening sometimes depending upon the severity of disease and internal physiological condition of the patient.

Clinical decision-making requires an accurate assessment of cardiovascular (CV) risk; benefits, hazards, and costs of management techniques must be balanced to determine the appropriate preventative measure for each individual (Loeser et al., 2013). Over the years, a variety of scores have been established to divide people into low, medium, and high CV risk groups. However, risk scores are not perfect, and comparing them side by side raises a lot of issues. Additionally, the small but significant difference between the anticipated and actual event rate leads to both under- and over-prediction of the disease complications and highlights the issue of validation (Miller and Revelo, 2018). The extrapolation to populations other than the original cohort, the choice to include traditional risk variables, the changes in population characteristics as a result of the time lag between observational studies and the application of risk scores, the exclusion of novel indices related to the disease pathophysiology are the factors associated with limitations of risk scores (Goldstein et al., 2015). Biomarkers are additional tools that can be used to further stratify a patient’s risk. A biomarker is described “a trait that is reliably tested and assessed as an indicator of normal biological processes, pathogenic processes, or pharmacologic responses to a therapeutic intervention” by the National Institutes of Health (Prasad K, 2015). In essence, cardiovascular disease biomarkers show early functional or morphological changes, long before the development of the overt disease especially in the context of prevention. This discovery of subclinical disease may create a window of opportunity for the prompt diagnosis and treatment of clinical CV disease (Saxena and Ng EYK, 2019). It has been demonstrated that a number of biomarkers reflecting these processes are useful in predicting the onset of symptoms and clinical outcomes in the pre and post aortic valve stenosis conditions (Bahler et al., 2018). Here, we systematically examine the biomarkers in the context of aortic stenosis and discuss their potential applications for risk stratification, which will ultimately help determine the extent of stenosis and further treatment wherever required.

## Material and methods

### Study samples

Patients above 20 years, suffering from complaints of shortness in breath, frequent fatigue, angina visiting the cardiology OPD at Dr. Ram Manohar Lohia Institute of Medical Sciences, Lucknow, Uttar Pradesh were enrolled in this study.

### Inclusion criteria

274 patients both male and female above 20 years, diagnosed and assessed with symptoms of CVD and recommended angiography by the cardiologists were included in this study. These patients were clinically assessed for the clarity of symptoms leading towards qualification of patients to be enrolled in this study. The criteria in clinical assessment are mentioned in the respective section below.

### Exclusion criteria

The patients having disorders such as chronic idney disease (CKD), surgery within 6 months, multiple organ disorders and below 20 years of age were excluded from the study.

Ethical Clearance for the study was provided by Dr. Ram Manohar Lohia Institute of Medical Sciences Institutional Ethical Committee. The study complies with all guidelines as mentioned during ethical clearance and informed consent was obtained from all the participants enrolled in the study.

In this study we have divided the 274 patients into three categories:

i) Mild/moderate patients with up to 20%–50% stenosis were grouped as mild/moderate and
ii) Severe patients with more than 50% stenosis in their vessels calculated by taking the average of stenosis in each of the five vessels LAD; Left anterior descending artery, Diagonal; Diagonal branches of left anterior descending coronary artery, LCX; Left circumflex, OM; Obtuse marginal (branch coronary arteries that come out from circumflex), RCA; Right coronary artery, PDA/PLV; posterior descending artery and posterior left ventricular artery. and as per the angiographic investigations.

### Clinical assessment

A structured medical history of all the patients with complaints like orthopnea; shortness of breath that occurs when lying flat, dyspnea; difficult or labored breathing while climbing or walking, edema in the lower body specially legs, weight loss, laziness and fatigue in response to diuretic treatment, complaints of mild, severe, consistent, occasional chest pain, known ischemic diabetes, chronic liver illness, chronic lung disease, high blood pressure, and heart disease visiting the cardiologists was taken with specific focus. Additionally, details about drinking patterns, drug use, and smoking behaviors were documented.

### Sample collection and processing

A total of 274 samples were collected from the subjects undergoing coronary artery angiography as advised by the cardiologist for evaluation of coronary artery disease. All the patients advised and admitted for angiography were subjected to the testing of some biochemical parameters. Samples were collected from these patients at the time of admission and 6 h after admission. Venous blood from these admitted cases were collected, centrifuged at 3,000 rpm and the supernatant serum was stored after centrifugation for evaluation of c-TnI, hsTn-I and hs-CRP. Plasma samples from fluoride vials were analyzed for Hemoglobin A1c (HbA1c), fasting plasma glucose (FPG), total cholesterol (TC), triglyceride (TG), high-density lipoprotein (HDL) cholesterol and low-density lipoprotein (LDL) cholesterol were analyzed enzymatically on Beckman Coulter automatic biochemical analyzer using commercial reagents. Serum Insulin concentrations were measured by electrochemiluminescence method. Some of the serum samples were kept for assessment of biochemical markers committed in this study. These parameters were used to identify the deviations leading to cardiac disorder and the level of stenosis in the subjects enrolled in the study. On the basis of the observations it was decided whether these biochemicals are normal or in the abnormal range for the patients and to what extent. Extent of carotid artery stenosis was estimated with the help of angiographic findings. Patients with upto 20%–50% stenosis were grouped as mild/moderate and more than 50% were grouped as severe stenosis as per the angiographic investigations.

### Biomarkers study

Serum cardiac Troponin-I (c-TnI) and Serum Creatinine, High sensitivity troponin I (hs-Troponin I) and High sensitivity C-reactive protein (hs-CRP) were analyzed in the collected samples both at the time of admission and 6 h after admission of the patients. The extent of mild and culprit stenosis was detected during angiographic examination and the same was reported in the form significant (>50% stenosis) and non-significant (<50% stenosis) Carotid Stenosis. On the basis of the extent of stenosis, the patients were categorized in three groups; normal, mild/moderate and severe and examined for the pattern of changes on the biomarker’s level. Patients with 10%–20% of stenosis were categorized as normal/control, those with 20%–50% of stenosis as mild/moderate and >50% as severe. The extent of stenosis was also investigated in all the five vessels LAD, LCX, RCA, OM, PDA/PLV as observed during angiography of the patients.

### Statistical analysis

The study employed One-way Analysis of Variance (ANOVA) followed by a *post hoc* analysis (Fischer LSD). This approach was employed to identify potentially significant parameters capable of discriminating between controls and patients with mild and severe stenosis. Correlation analysis was performed to visualize the relation between different features in the normal, mild and severe stenosis groups of patients and the changing level of markers associated with the stenosis (if any). Principle component analysis (PCA) test was used to understand the clustering of healthy controls, mild stenosis and severe stenosis groups.

## Results

**FIGURE 1:**
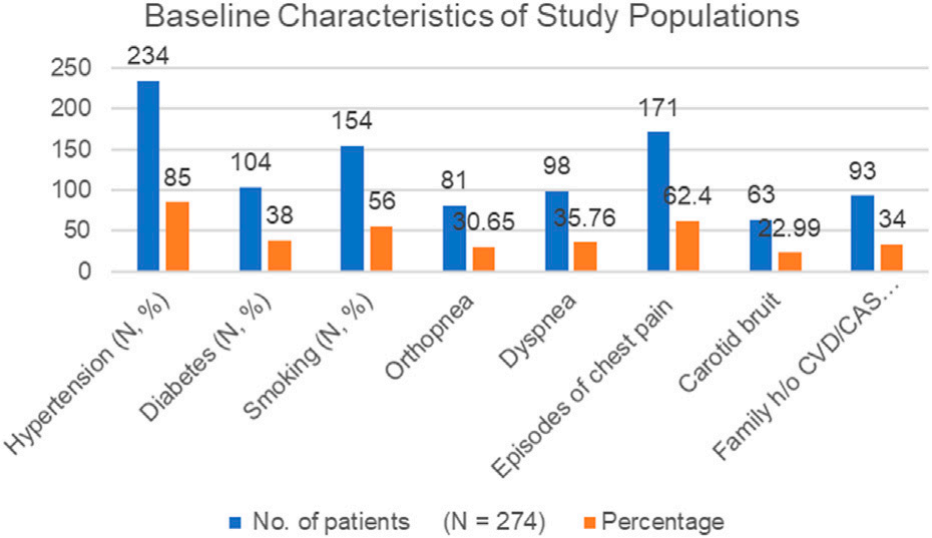
Baseline characteristics of the study population.

Figure 1 shows the baseline characteristics of the study population. As observed, out of 274 study subjects, 187 were male and the remaining 87 were female, out of which 85% (234) subjects were found to have hypertension and 104 (38%) were diabetic. 154 subjects (56%) had smoking habits. 81 patients (30.65%) had problems of orthopnea; shortness of breath that occurs when lying flat, 98 (35.76%) had dyspnea; difficult or labored breathing when walking or climbing stairs. 171 patients (62.40%) were detected with sporadic episodes of chest pain and 63 patients (22.99%) were diagnosed with carotid bruit (vascular sound that is usually heard with a stethoscope over a carotid artery). 56% (154) had smoking habit and 27% of the study population had a history of coronary artery stenosis/cardiovascular disorder (Figure 1).

Table 1 shows the baseline clinical characteristics of the study population. We investigated the basic clinical like fasting blood glucose, total cholesterol, HDL, LDL, serum urea and serum creatinine of the study subjects. Fasting blood glucose is done to detect the diabetic status of a patient, Total cholesterol, HDL, LDL is done to investigate the hyperlipidemic status of a patient. Creatinine and urea blood levels reflect glomerular filtration rate. Serum creatinine is used as a gauge of renal function and, particularly in men, shows a U-shaped relationship with CAD. Thus, both low and high glomerular filtration rates are associated with a higher risk of developing diabetes and coronary artery disease (CAD). These biochemical parameters were studied to find the extent of coronary artery disease in the subjects enrolled. We observed that most of the patients in our study were found to be diabetic and dyslipidemia with higher levels of total cholesterol and LDL. Some of them had increased levels of serum urea and serum creatinine suggesting that these may be contributing to CAD to certain extent in these patients.

**TABLE 1:** Basic clinical characteristics of the study population.

Variables	Values (Mean ± SD) (N = 274)	Reference range
Gender (Age): Male/Female	187 (58.94 ± 11.56), 87 (56.89 ± 11.15)	
Fasting blood glucose	138 ± 34.89	90–100 mg/dL
Total cholesterol	263 ± 56.72	<200 mg/dL
HDL	35.89 ± 7.42	35–65 mg/dL for men, 35–80 mg/dL for women
LDL	139 ± 9.63	<100 mg/dL
Serum Urea	42.45 ± 16.41	5–20 mg/dL
Serum Creatinine	1.68 ± 1.07	0.7–1.3 mg/dL for men and 0.6–1.1 mg/dL for women

Fasting blood glucose, total cholesterol, HDL, LDL, serum urea and serum creatinine all were measured in mg/dL. Here these values are expressed as Mean ± SD.

Table 2, shows the degree of coronary artery stenosis (as evident from the angiographic findings) in the study population. Here the patients with no stenosis (those suffering from sporadic episodes of angina due to certain level of myocardial necrosis) are treated normal. Those with 20% to <50% stenosis in the arteries are categorized in the non-significant group while those with >50% stenosis are considered as significant. Table 2 represents the number of patients with hypertension and diabetes that is highest in the significant stenosis group. Most of the patients in this group are smokers and had increased creatinine level as compared to the normal and non-significant stenosis category. The serum urea and serum creatinine levels were found to be almost equal in the normal and non-significant stenosis group.

**TABLE 2:** Comparison of patient characteristics according to the degree of Carotid artery Stenosis.

Variables	Control; Normal Carotid Artery (*n* = 36)	Mild; Non-significant CAS (<50%) *n* = 27	Severe; Significant CAS (>50%) *n* = 210
Age years	58.703 ± 12.75	63.70 ± 6.94	57.87 ± 11.51
Hypertension	29	20	128
Diabetes	18	17	97
Smoking	29	24	144
Serum Urea	16.28 ± 1.73	25.36 ± 1.22	42.33 ± 3.54
Serum Creatinine	1.004 ± 0.16	0.98 ± 0.24	1.89 ± 1.01

**FIGURE 2:**
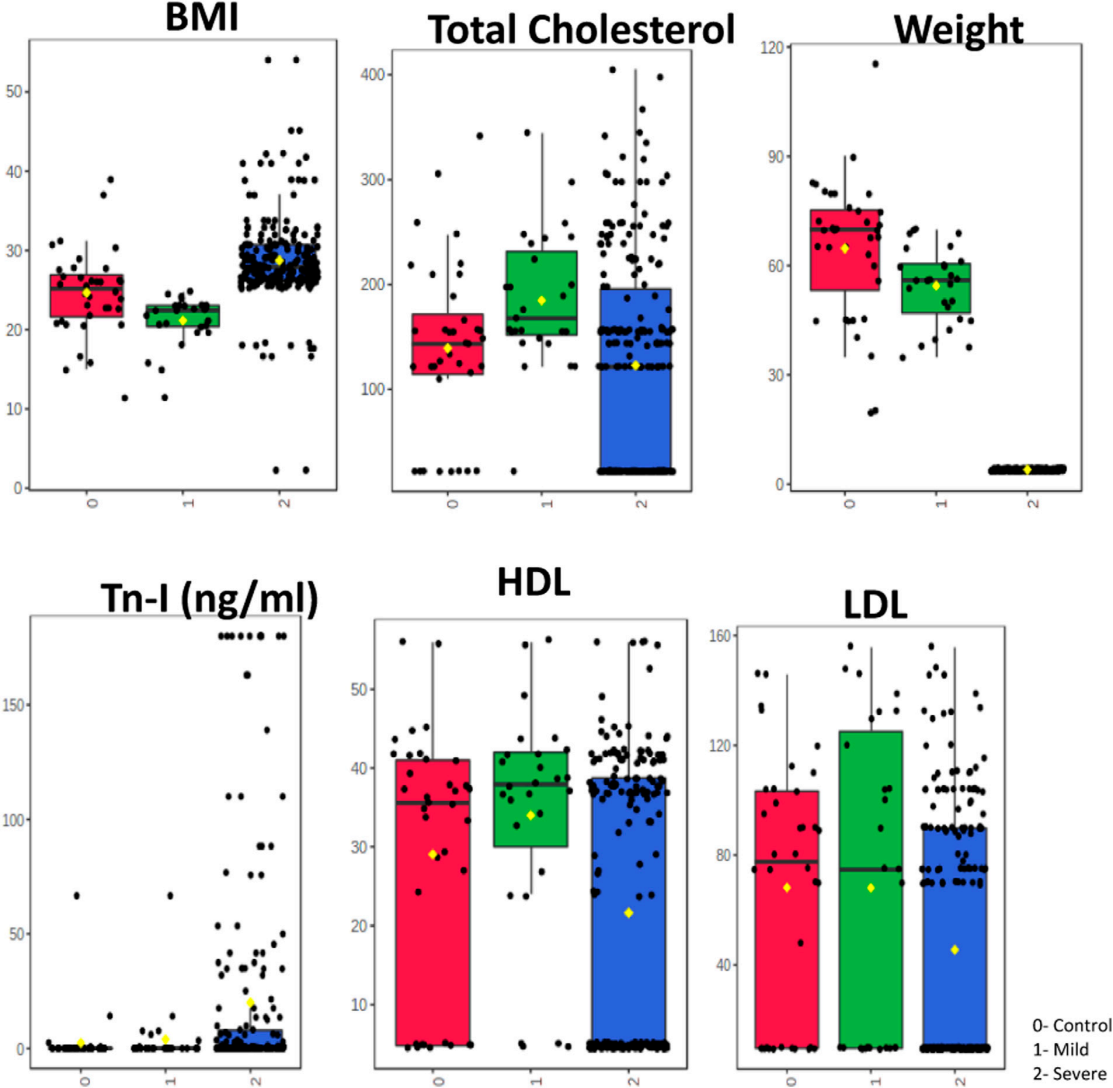
One-way Anova. One-way Analysis of Variance (ANOVA) was employed to investigate the profile of different parameters such as BMI, Weight, Tn-I, total cholesterol, HDL, LDL, among patients with carotid artery. ANOVA was followed by a *post hoc* analysis (Fischer LSD) to identify potentially significant parameters that can discriminate between controls, mild and severe.

**FIGURE 3:**
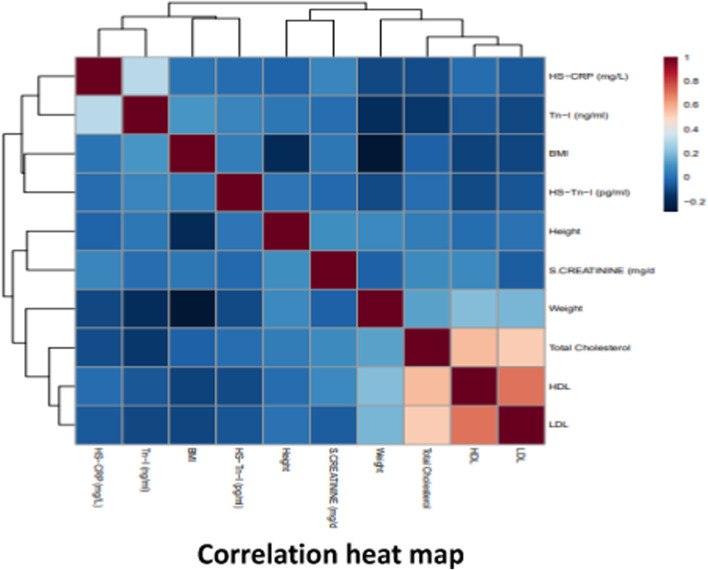
Correlogram of ten parameters with clustering analysis performed in Metaboanalyst 5.0. Data was analysed using Pearson correlation analysis. The scale shown on the right side (red and blue colors indicate positive and negative correlation). Correlation analysis to visualize the relation between different features in the normal, mild and severe stenosis groups of patients.

As observed in the results of one-way analysis of variance (Figure 2), BMI was found to be highest in the patients with severe stenosis, i.e., with more than 50% blockage. Total cholesterol was highest in both mild and severe category. While LDL cholesterol level was found to be elevated in all the three groups but was highest in the mild group. Troponin I was higher in the severe stenosis category. Figure 3 shows a correlation heat map within the variables used in this study among the three categories of patients. As seen in the map, the lower right corner represents a strong correlation between the variables in all the categories and it is found that total cholesterol, LDL and HDL cholesterol levels correlate strongly in a positive manner with each other. Cholesterol levels always pose a threat of vascular stenosis and hence could render a possibility of cardiovascular disorder arising due to hypercholesterolemia condition in these patients. Thus Figures 2, 3 make it very obvious that uncontrolled BMI, increasing weight and hyperlipidemia conditions are prominent reasons of vascular stenosis in cardiovascular disorder patients.

**FIGURE 4:**
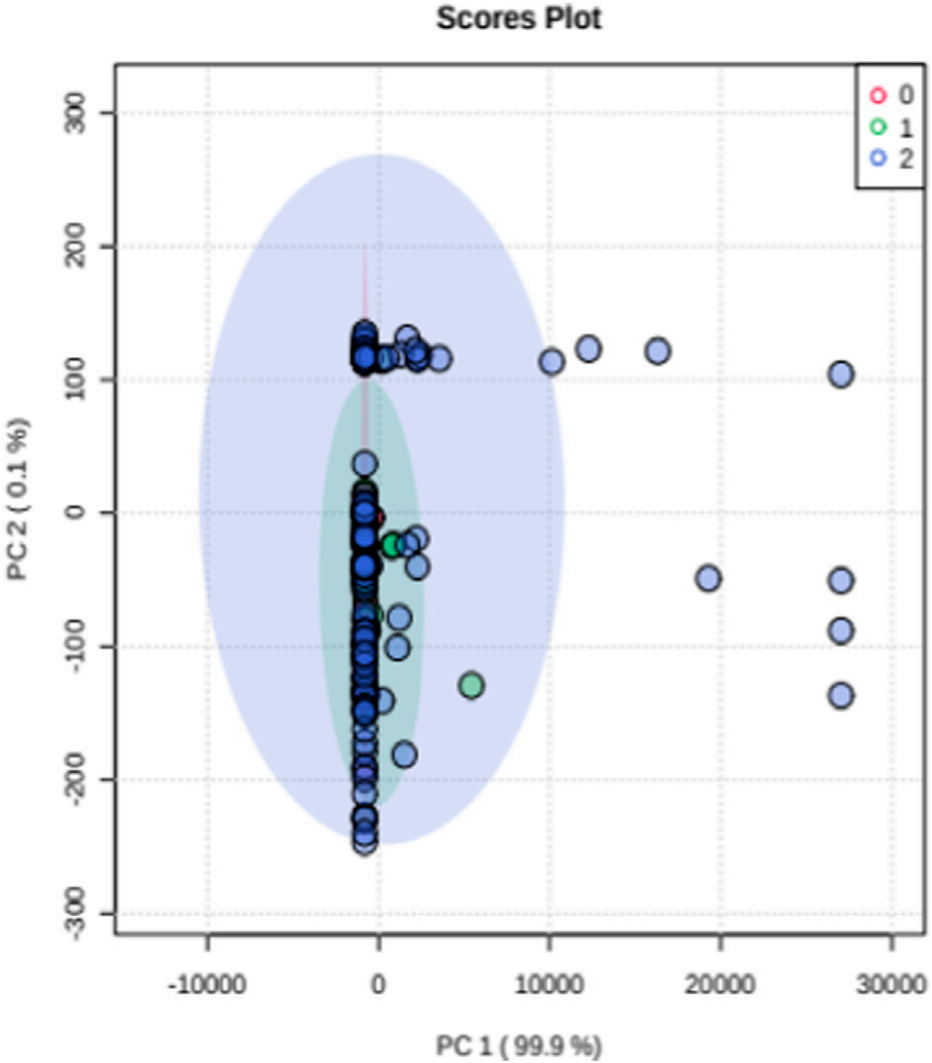
Principle component analysis (PCA).

**FIGURE 5:**
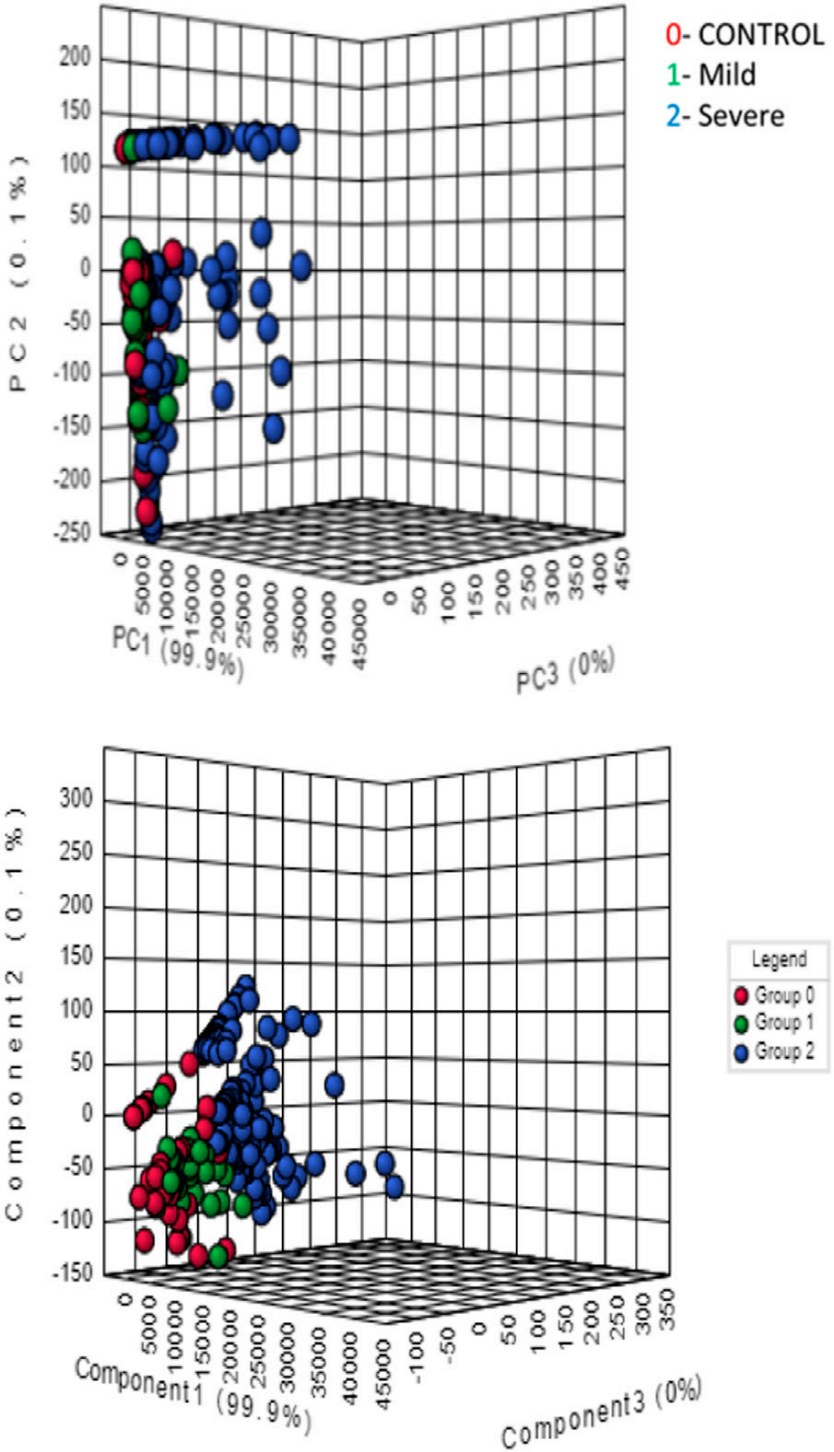
Partial Least square determinant analysis (PLS-DA).

Principle component analysis (PCA) test was used to understand the clustering of healthy controls, mild stenosis and severe stenosis groups (Figure 4). Separation of the three groups was found to be less evident. We further performed Partial least square determinant analysis (PLS-DA) that showed good separation of the three groups and cross validation resulting in good model parameters (R2 = 0.86163, Q2 = 0.85006) (Figure 5).

**FIGURE 6:**
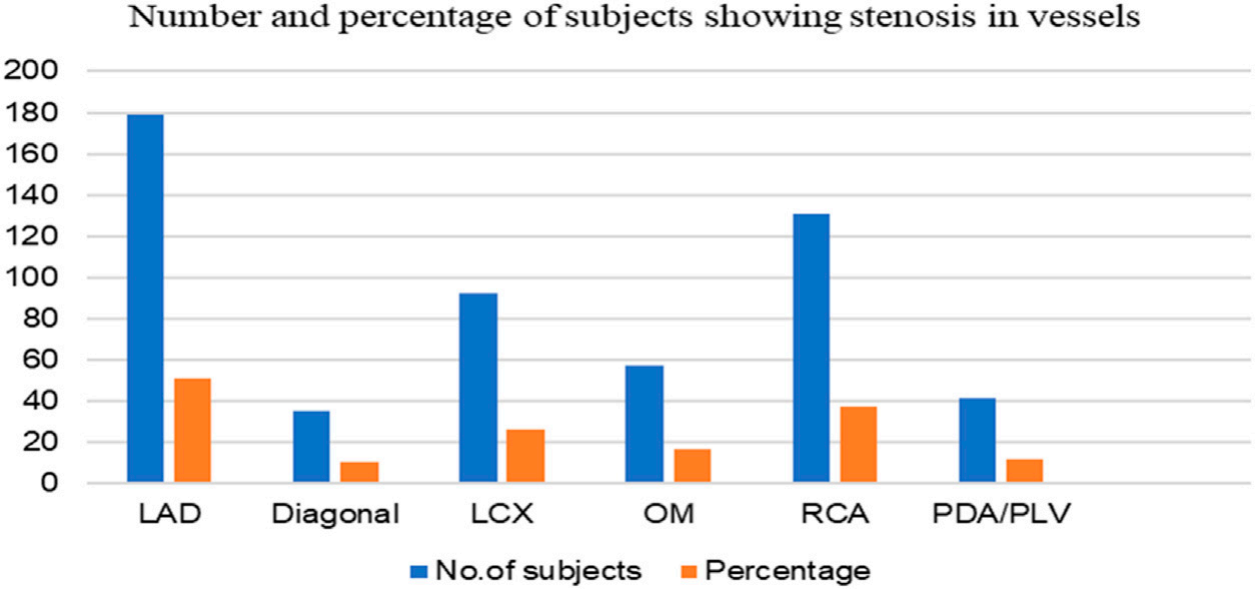
Number and percentage of study subjects showing stenosis in the different vessels. LAD, Left anterior descending artery; Diagonal, Diagonal branches of left anterior descending coronary artery; LCX, Left circumflex; OM, Obtuse marginal (branch coronary arteries that come out from circumflex); RCA, Right coronary artery; PDA/PLV, posterior descending artery and posterior left ventricular artery.

Figure 6, represents the number of subjects having different extent of stenosis in their vasculature. For this we have considered the level of stenosis in six different vessels; LAD, Diagonal, LCX, OM, RCA and PDA/PLV. We found that most of the subjects (42%) had stenosis in their LAD followed by 38% who had stenosis in their RCA. Almost 23% patients were reported to have stenosis in their LCX followed by OM (18% patients), PDA/PLV (13%) and only 10% patients had blockage problem in their diagonal.

Table 3 represents the clinical characteristics of the study population. Out of the 274 study subjects, 186 were reported to have culprit stenosis (>90% stenosis in the reported arteries). 24% of the subjects were found to have stenosis in a single vessel and hence were categorized in the Single Vessel Disease (SVD) group while 76% were having stenosis in two or more than two arteries (Multiple Vessel Disease). As per the ECG findings of the subjects in this study, 164 (59%) of the study subjects were found to have STEMI (ST elevation myocardial infarction) while 40% (110) were NSTEMI (non-ST elevation EMI). Out of the 186 cases with Culprit stenosis, 139 (75%) had STEMI. 106 (40%) cases with MVD had STEMI and 60% (161) cases had NSTEMI. 70% (58) cases with SVD had STEMI while the remaining 30% (25) cases had NSTEMI. 143 cases (87%) out of 164 STEMI had shown T-wave inversion while 21 (13%) had no changes in their T-wave. 103 cases showing T-wave inversion were reported to have culprit stenosis and 90 cases were having MVD.

**TABLE 3:** Clinical Characteristics of the study groups.

Predictors	No. of patients	Culprit stenosis	Single vessel	Multiple vessel
Overall	274	186	83	191
ECG (ST Elevation)				
STEMI	164	139	58	106
NSTEMI	110	47	25	38
ECG -T-wave inversion (in STEMI group)				
Yes	143	103	53	90
No	21	36	05	16

Table 4 shows the changing pattern of hs-CRP, c-TnI and hs-TnI in all the three classes of stenosis patients. These markers were also studied in samples collected at two different time intervals to see the change in their level in a time dependent manner after the onset of chest pain or any symptom which caused the basis of the patient’s admission for angiography. As observed, the level of hs-CRP kept increasing with time and severity of stenosis in all the three categories of patients. Hs-CRP is a potent inflammation marker and tends to increase with the severity in diseased conditions until the root cause of the problem/disease is taken care. Cardiac troponin I is a universal marker of CVD and has been utilized here to see it’s correlation with the severity of stenosis but as evident, cTnI doesn’t show any change at the initial stages of stenosis in the vessels. Also, it could be measured only after 6–12 h in mild stenosis patients (as shown in Table 4). C-TnI tend to change significantly in the severe stenosis patients after 6 h. On the other hand high sensitivity Troponin I also didn’t change in normal healthy subjects as it could not be detected in their samples but shows a prominent change in both mild and severe stenosis groups both at the admission and after 6 h of admission when the patients were being taken for angiography. These observations show that as compared to c-TnI, hs-TnI proves to be analysed more efficiently in the sample of patients both in a severity and time dependent manner.

**TABLE 4:** Changing level of serum markers with respect to the time and severity of stenosis in subjects enrolled for the study.

Category of stenosis patients	Control; Normal Carotid Artery (*n* = 36)		Mild; Non-significant CAS (<50%) *n* = 27		Severe; Significant CAS (>50%) *n* = 210		Reference range
Time of sample collection Parameters	T0	T1	T0	T1	T0	T1	
hs-CRP (mg/L)	0.068	0.072	1.32	2.79	3.46	7.81	0.3–10 mg/L
c-TnI (ng/mL)	ND	ND	ND	0.031	1.82	4.89	0 and 0.04 ng/mL
hs-TnI (ng/mL)	ND	ND	1.026	3.998	7.362	19.416	0.024 ng/mL to 0.03

T0, Time of admission (onset of chest pain or any CAD, clinical symptom diagnosed by clinician); T1, 6 hrs after admission; ND, Non-detectable values.

## Discussion

The carotid artery (CA) bifurcation or the internal CA are more likely to develop carotid artery (CA) stenosis, which is brought on by localised thickening of the CA wall owing to atherosclerosis. Significant CA stenosis is said to affect 7%–9% of the general population (Arsenault et al., 2014). The high frequency is linked to atherosclerosis (11%) and coronary heart disease (18%) as well as acute ischemic stroke (60%), according to a study (Tan et al., 2022). At least 20% of all ischemic strokes are brought on by thromboembolisms from CA atherosclerotic plaque (Abd alamir et al., 2019). Dyslipidemia, hypertension, smoking, diabetes, and several hemodynamic characteristics, such as low wall shear stress or turbulent blood flow, all accelerate the development of CA atherosclerosis (Chou et al., 2014). A growing body of research suggests that patients with symptomatic CA stenosis of at least 70% should have quick surgery, and the majority of recommendations call for carotid endarterectomy (CEA) within 2 weeks of mild ischemic strokes or transient ischemic episodes (TIAs). Contrarily, some guidelines also advise CEA for asymptomatic CA stenosis, but these recommendations are less strong than those for surgical or interventional procedures (Scott et al., 2015). Number of factors contribute to the extent of stenosis and related complication. Few prominent ones explained (Colombo and Latib, 2012) and also targeted in this study such as weight, BMI, obesity, total cholesterol, glucose, urea, and creatinine have been found to be associated with the mechanism and extent of stenosis leading to ST elevations and T-wave inversions in the patients targeted in this study.

The surprising thing is that the period of time when the risks of ischemic stroke with CA stenosis decreased coincided with the expansion of intensive modern medical care, including the diagnosis and management of stenosis at a very early stage with the help of suitable markers and drugs.

### Weight, BMI and obesity in vascular stenosis

The association between lipid abnormalities and obesity is established through the progression of insulin resistance in peripheral tissues. This leads to an augmented hepatic influx of fatty acids originating from dietary sources, intravascular lipolysis and adipose tissue that is unresponsive to the antilipolytic effects of insulin. Obesity is a chronic metabolic condition linked to CVD, increased morbidity, and mortality (Klop and Elte, 2013). It is often characterized by increased weight, increased BMI, hypertension, and dyslipidemia. Together, these elements influence the production of plaque, which leads to stenosis (Kojta and Chacińska, 2020). It is clear that the culmination of all these modifications and adaptations in the arteries supplying the heart causes CVD. Our observations are in agreement with these investigators (Tables 1, 2; Figure 3)

### Total cholesterol, HDL, LDL in vascular stenosis in hypertension and vascular stenosis

Coronary atherosclerosis can develop and occur for a variety of reasons. Dyslipidemia promotes and exacerbates coronary atherosclerosis as a known risk factor. A growing body of research suggests that the advancement of stenosis and the development of CVD may be aided by the decline in HDL-C and the rise in LDL-C (Upadhyay RK, 2015). An independent predictor of atherosclerotic cardiovascular disease is an elevated LDL-C level. Reduced LDL-C levels can lower the risk of cardiovascular disease and events such ischemic stroke and AMI (Sun et al., 2022). Increased levels of HDL-C, as opposed to LDL-C, have been shown to successfully decrease atherosclerosis and lower the incidence of disorders connected to atherosclerosis (Harreiter J, 2019). Increases in total and LDL cholesterol can also cause endothelial damage in the arteries, making it easier for atherosclerotic plaque to develop and cause stenosis on the arterial wall and thrombosis in CVD (Kojta and Chacińska, 2020). Although high levels of HDL-C can strengthen the tissue that surrounds the artery wall and reduce stenosis on the endothelium surface, low levels of HDL-C are less effective in removing cholesterol from the body and can hasten the development of atherosclerosis. TC, HDL-C (inversely), hypertension, smoking, and hyperglycemia have all been found to be independent risk factors for the development of CA stenosis (von Birgelen et al., 2003). In accordance with these researchers, we also discovered greater levels of total cholesterol and LDL cholesterol, as well as a lower amount of HDL cholesterol, in the patients with mild and severe stenosis (Table 1; Figure 2).

### Glucose, urea and creatinine in vascular stenosis

High levels of serum creatinine and serum urea are known to be associated with hyperglycemia and vice-a-versa. High urea and creatinine are prominent biomarkers of kidney disorders and with high glucose content it becomes evident that kidney dysfunction due to diabetic condition contributes to the excess of these toxins in blood (Sun et al., 2022). Since hyperglycemia is also known to be associated with atherosclerosis and the latter is a process in stenosis, it could be said that these four factors: urea, creatinine, glucose and atherosclerosis leads to vascular stenosis that further causes cardiovascular disorders (Baldwin et al., 2011) (Tables 1, 2; Figure 3).

### Stenosis, ST elevations and T-wave inversions

Variations in the ST- and T-waves may signify heart disease or be a typical variety. The clinical setting and the existence of similar abnormalities on earlier electrocardiograms, therefore, play a role in how the results should be interpreted. An elevation in the ST segment indicates a complete blockage of the heart’s major arteries, often associated with heart attacks caused by the hindrance of blood flow due to the plaque buildup (Sun Z, 2013). The primary cause of most heart attacks is the accumulation of fatty, waxy plaque within the arteries. A blood clot forming on this plaque closes the artery, plummeting blood supply to the heart muscle. Ischemia, resulting from partial or complete blood flow blockage, leads to inadequate blood supply to the heart muscle during a heart attack, causing worsening in the ventricular muscle (Adjedj et al., 2017). Complications such as cardiogenic shock can come about from muscle damage, affecting the heart’s pumping function. Severe electrical rhythms like ventricular tachycardia or ventricular fibrillation can lead to cardiac arrest and sudden death. STEMIs are frequently heart attacks that are more serious.

In STEMI, T-wave inversion serves as a predictive indicator of substantial coronary artery stenosis and pinpoints a subgroup with an unfavorable prognosis when undergoing medical treatment (Zhang and LinMu, 2018). Thus, both STEMI and T-wave inversions signifies stenosis in the vessels supplying the heart and their elevations and inversions denote the extent of stenosis. In our study (Table 3) we have found more patients with culprit stenosis (more than 50%) in multiple vessels.

### hs-CRP, c-TnI and hs-TnI in stenosis

The presence of macrophages and T cells in plaque, which eventually induce stenosis, and its association with the phenomenon of instability, which can result in the development of an ischemia event, may be related to elevated hs-CRP levels (Wang et al., 2021). Therefore, in patients with high-grade carotid stenosis, hs-CRP levels are considered a valuable supplementary risk measure (Liang et al., 2015). In line with our findings (Table 4) plasma troponin concentrations are thought to be a highly specific diagnostic for myocardial damage. On the actin filament, the troponin complex which consists of three subunits; troponin I, T, and C along with tropomyosin is crucial for the calcium-mediated control of skeletal and cardiac muscle contraction. 1 Troponin I, T, and C each have tissue-specific isoforms (Hayashi et al., 2008). The C subunit of troponin doesn’t specifically belong to myocardium and is therefore not employed in assays for the diagnosis of heart damage. In myocardial tissue, there is only one cardiac troponin I (cTnI) isoform. The N-terminus of this isoform features a 32 amino acid post-translational tail. The creation of highly specific monoclonal antibodies that does not show any cross-reactivity with other substances was done in such a manner that it shows less than 50% similarity with the other two isoforms (Tveit et al., 2022). Cardiac troponin concentrations begin to rise only after 6 h from the onset of symptoms. Thus, could be detected in the sample only after 6–9 h which means samples should be collected twice; at the time of admission and 6–9 h later than the first collect. Peak values of cTnI appears after 18–24 h of symptom onset (Wu et al., 2022). Our observation regarding the peaking time of cTnI and hs-TnI in correlation to the extent of stenosis (Table 4) is in agreement with the findings of these investigators.

Cardiovascular troponin I (cTnI) and cardiac troponin T (cTnT) stand out as the preferred biomarkers for identifying myocardial damage due to their exceptional sensitivity and specificity as cardiac indicators. Recent papers include practical clinical considerations in the assessment of cTn increases and guidelines for the use of cTn measurement in acute cardiac care (Chin et al., 2014). High-sensitivity (hs)-cTn assays, a new generation of cTn diagnostics, have recently been adopted into accepted clinical practise (Tveit et al., 2022). The analytical sensitivity of cTn tests has gradually improved over time. These assays measure the same analyte as did prior generations of assays, but at modest measurement ranges with noticeably improved analytical sensitivity. It is also important to note that, despite how assays are named by manufacturers, hs-cTn assays should only be designated as hs-cTn assays if they meet the below listed analytical characteristics and have also been published in peer-reviewed literature. This is because there are differences in routine use. From a clinical perspective, advancements in analytical performance seen in high sensitivity cardiac troponin assays have significantly enhanced their ability to identify subtle variations in cardiac troponin concentrations during sequential testing. This improved sensitivity also enables the detection of minimal amounts of myocardial damage, surpassing the capabilities of earlier cTn assay generation (Hayashi et al., 2008). It is expected that the proper utilization of hs-cTn assays will enhance early diagnosis and contribute to effective short- and long-term risk stratification.

### Novelty and implications

The incorporation of hs-TnI and hs-CRP in combination at an early stage of CVD diagnosis will enable early detection of myocardial injury, facilitating timely intervention and prevention of adverse cardiovascular events. This will enhance the capability of the medical fraternity in designing risk stratification algorithms, enabling more accurate assessment of cardiovascular risk and personalized treatment strategies tailored to individual patient profiles. This will help in enhanced prognostic insights, aiding in the prediction of disease progression, response to therapy, and long-term outcomes. Early detection of the biomarkers as compared to the c-TnI will also help in the management of losses associated with the exaggeration of complexities seen in CVD.

### Limitations of the study

Human subjects-based study always is limited in terms of follow-up of patients leading to unintentional loss of data. This demands the investment of time and high patient number at the initial stage which can compensate the loss. The inferences drawn in this study could be utilized to conduct an extensive study with a greater number of patients. Also, the biomarkers focused in this study can be examined in the follow-up patients even after angiography and angioplasty to see the pattern of these markers in the course of CVD/CAD.

## Conclusion

CAD is mainly a neglected lifestyle diseases signified by various extents of stenosis in the major vessels supplying to/from the myocardium. If the extent of stenosis or the problems associated with CAD could be diagnosed pretty earlier with the onset of the diseases, the complications associated could be mitigated well in advance thereby reducing the waste of time, economy, and energy of the patient. Changing pattern of biomarkers can also save the patient from the trauma of getting through the invasive procedures of angiography, angioplasty and Carotid endarterectomy. cTnI gets detected in the patients only after 12 h while hs-TnI could be detected within 6 h. High-sensitivity troponin assays show major potential as a prospective clinical tool for individuals with CAD and CAS condition.

## Data Availability

The original contributions presented in the study are included in the article/Supplementary Material, further inquiries can be directed to the corresponding authors.
